# Multidisciplinary View of Alcohol Use Disorder: From a Psychiatric Illness to a Major Liver Disease

**DOI:** 10.3390/biom6010011

**Published:** 2016-01-15

**Authors:** Stefano Gitto, Lucia Golfieri, Fabio Caputo, Silvana Grandi, Pietro Andreone

**Affiliations:** 1 Department of Gastroenterology, Azienda Ospedaliero-Universitaria & University of Modena and Reggio Emilia, Modena 41124, Italy; stefano.gitto@studio.unibo.it; 2 Department of Psychology, University of Bologna, Bologna 40138, Italy; lucia.golfieri@gmail.com (L.C.); silvana.grandi@unibo.it (S.G.); 3 Department of Internal Medicine, SS Annunziata Hospital, Cento, Ferrara 44011, Italy; f.caputo@ausl.fe.it; 4 “G. Fontana” Centre for the Study and Multidisciplinary Treatment of Alcohol Addiction, Department of Clinical Medicine, University of Bologna, Bologna 40138, Italy; 5 Department of Medical and Surgical Sciences, University of Bologna and Azienda Ospedaliero-Universitaria di Bologna, Policlinico Sant’Orsola Malpighi, Via Massarenti 9, Bologna 40138, Italy

**Keywords:** alcohol use disorder, alcohol liver disease, liver transplantation

## Abstract

Alcohol use disorder is a significant health problem being a cause of increased morbidity and mortality worldwide. Alcohol-related illness has a relevant economic impact on the society and a negative influence on the life of patients and their family members. Psychosocial support might be useful in the management of people affected by alcohol use disorder since psychiatric and pharmaceutical approaches show some limits. In fact, many drugs are accessible for the treatment of alcohol disorder, but only Baclofen is functional as an anti-craving drug in patients with advanced liver disease. The alcohol-related liver damage represents the most frequent cause of advanced liver disease in Europe, and it is the main cause of death among adults with high alcohol consumption. The multidisciplinary action of clinical-psychologists, psychiatrics and hepatologists, is essential in the management of patients with alcohol liver disease especially in the case of liver transplantation. In general, the multidisciplinary approach is necessary in prevention, in framing patients and in the treatment. More resources should be used in prevention and research with the main aim of decreasing the harmful alcohol consumption.

## 1. Introduction

Production and consumption of alcohol represent a relevant part of the economy and culture of many countries worldwide. Alcoholic beverages are consumed during ordinary recreational activities, and it is well known that their intake can decrease both anxiety and inhibition. At the same time, alcohol-related morbidity and mortality is a major problem for public health. The clinical consequences of alcohol ingestion vary according to the extent and method of habit and differ with environmental and individual factors. In the United States, alcohol use is associated with 50% of liver-related deaths, accounting for a health cost of about $3 billion annually [1]. Interestingly, in the last few years, the fastest annual increase in alcohol consumption (400%) has been registered in the People’s Republic of China, perhaps because of its great and rapid economic growth [2]. Concerning Europe, in 2006, the Institute of Alcohol Studies reported that it is the region with the heaviest drinking, with 23 million people who develop an alcohol-related disorder every year [3]. In this area, the prevalence of alcohol consumption varies from 0.1% to 6.6% [4] but overall, 11% of deaths in men and 1.8% in women are connected to alcohol use [5].

The United States National Institute on Alcohol Abuse and Alcoholism defined “heavy” drinking as ingesting more than four units in a day or 14 drinks in a week for males, and three drinks or seven drinks for females. Remarkably, about a quarter of heavy drinkers may develop alcohol-related health problems [6]. Notably, a meta-analysis [7] clearly showed that light drinking (up to one drink per day) increases the hazards of getting cancer of the oral cavity, pharynx, esophagus, and female breasts.

The relevant economic impact of the alcohol use has to be taken into account too. In Europe, the annual direct cost related to alcohol, ranges from €1 billion to €7.8 billion. This specific expense accounts for 0.04%–0.31% of annual gross domestic product [8]. Notably, 61% of the expenses associated with alcohol use can be ascribed to heavy drinking [9].

With this review, we tried to analyze the psychiatric and psychological aspects of alcoholism, alcohol-related liver disease, and, finally, the common land represented by the liver transplantation (LT). The literature search included published articles (peer reviewed original, review and meta-analyses). The search terms included “alcohol use disorder”, “alcoholic liver disease”, and “alcohol and liver transplantation”.

## 2. Definition and Terminology

The terminology surrounding alcohol dependence, alcohol abuse and alcohol misuse is often ambiguous. In 2013, the American Psychiatric Association published the fifth edition of Diagnostic and Statistical Manual of Mental Disorders (DSM-5) changing the classification of alcohol use [10]. Although there is substantial overlap between DSM-5 and the previous version (DSM-IV “Text Revision”), some important differences can be found. Concerning the terminology, DSM-IV “Text Revision” defined two distinct conditions: the alcohol abuse and the alcohol dependence. The last edition integrates these two conditions into a single one called alcohol use disorder (AUD). Indeed, AUD can be classified in three subgroups: mild, moderate, and severe. In the DSM-IV “Text Revision” era, the diagnostic criteria for abuse and dependence were distinct. Patients who meet one or more of the “abuse” criteria within a 12-month period would receive the “abuse” diagnosis while subjects with three or more of the “dependence” criteria would receive a “dependence” diagnosis. On the contrary, with the DSM-5, the definition of AUD may be satisfied if at least two of the eleven diagnostic criteria are present (see all the criteria in Table 1). The presence of two or three symptoms indicate a mild disorder, four or five symptoms a moderate one, and six or more symptoms a severe disorder. Notably, DSM-5 removes “legal problems” between the diagnostic criteria adding the craving [11].

**Table 1:** The fifth edition of Diagnostic and Statistical Manual of Mental Disorders (DSM-5) criteria for the framing of patients with alcohol use disorder (AUD).

1. Alcohol is often taken in larger amounts or over a longer period than was intended.
2. There is a persistent desire or unsuccessful efforts to cut down or control alcohol use.
3. A great deal of time is spent in activities necessary to obtain alcohol, use alcohol, or recover from its effects.
4. Craving, or a strong desire or urge to use alcohol.
5. Recurrent alcohol use resulting in a failure to fulfill major role obligations at work, school, or home.
6. Continued alcohol use despite having persistent or recurrent social or interpersonal problems caused or exacerbated by the effects of alcohol.
7. Important social, occupational, or recreational activities are given up or reduced because of alcohol use.
8. Recurrent alcohol use in situations in which it is physically hazardous.
9. Alcohol use is continued despite knowledge of having a persistent or recurrent physical or psychological problem that is likely to have been caused or exacerbated by alcohol.
10. Tolerance, as defined by either of the following: (a) A need for markedly increased amounts of alcohol to achieve intoxication or desired effect; (b) A markedly diminished effect with continued use of the same amount of alcohol.
11. Withdrawal, as manifested by either of the following: (a) The characteristic withdrawal syndrome for alcohol (refer to criteria A and B of the criteria set for alcohol withdrawal); (b) Alcohol is taken to relieve or avoid withdrawal symptoms.

A substantial heterogeneity can be found among patients with AUD regarding age of onset, kind of drinking (continuous or binge), alcohol metabolism (worst in females) and presence or absence of associated psychiatric disorder [12]. With the aim of providing targeted treatments and establishing the correct prognosis, it is mandatory to adequately evaluate the patients [13]. In the last few years, the concept underlying the identification of AUD typologies has evolved. AUD classification can be conceived as a disease state (Jellinek gamma and delta), as a personality model supported by genetic substrate (Cloninger type I and type II) [14], and as a measure of disease severity determined through cluster analysis (Babor type A and type B) [15]. Cloninger’s classification is the most utilized among clinicians. Cloninger’s type I patients often develop AUDs late during the life after traumatic life events, have relief craving, and tend to evade risky situations (harm avoidance) [16]. These patients show a good response to a “light” treatment approach aimed at improving their “coping skills” without alcohol consumption. In some cases, a short-term residential staying combined with self-help groups may be helpful [14]. Cloninger’s type II patients show a higher genetic component, develop AUDs usually early in life, have a personality disorder or other psychiatric co-morbidities, show a reward craving, and tend to seek new emotional situations (novelty seeking) [16]. These subjects need a more intensive and continuous approach by a multidisciplinary team. Medical, psychological and social support would be opportune, and a medium-long term residential intervention may often be necessary [17].

Patients affected by AUD can be classified also according to the craving pattern [18]. Alcohol craving plays a critical role in AUD influencing the pharmacological choices and the main predictors of alcohol recidivism [19]. In details, we have the following subtypes: (a) “reward craving” (often accompanied by family history of alcoholism, characterized by dopaminergic/opioidergic deregulation and characteristic personality trait defined by the search of reward); (b) “relief craving” (GABAergic/glutamatergic deregulation, significant reactivity to stress, withdrawal symptoms, and reactive drinking); (c) “obsessive craving” (serotoninergic deregulation, personality traits consisting of absence of inhibition, loss of control, compulsive drinking, and alcohol-related damage) [20]. Interestingly, the Craving Typology Questionnaire is a not yet validated diagnostic tool able to select patients according to their typology of craving [21].

Remarkably, the binge drinking represents a specific alcohol consumption pattern. The National Institute on Alcohol Abuse and Alcoholism defines it as the consumption of five or more drinks (male) or four or more drinks (female) in about 2 h [22]. The phenomenon of binge drinking is growing in Western countries, and is particularly striking in beer- and spirit-drinking cultures in the UK and northern Europe [23]. Between 1993 and 2001 in the USA, binge-drinking events per year increased by 17%, with the highest rates occurring among 18 and 25 years [24]. In the UK, the Health Survey for England reported that 57% of young males were binge drinkers [25] and similar percentages can be found in all parts of Europe [26].

## 3. Social Impact and Psychological Approach

One of the main social and medical problems regarding the AUD derived by the widespread opinion that it is not a disease but a fault. This judgment can win in the public opinion as well as among medical professionals [27]. In the last few years, our knowledge about the alcoholism has been greatly increased thanks to both informative campaigns and education, and today we can assert that alcoholism is really a disease and not a moral problem [28].

AUD is a clinical condition associated with substantial disability and loss of quality of life [29]. Furthermore, alcohol use is a major reason of accidents and violence episodes [30]. Singh *et al.* [31] recently proposed a study to assess the prevalence of violence among young males and females with AUD and seeking emergency department care. Among 842 patients, a quarter reported dating violence in the past year. Major risk factors were female gender, Caucasian race, receipt of public assistance, older age of drinking onset, suicidal ideation or attempt. Given these results, authors suggest that the public health policies should be targeted especially towards the young people [32]. Several methods have been used to decrease binge drinking. They include policies to reduce availability of alcohol by limiting the hours and places of sale, to create minimum age purchase laws and to raise the price of alcohol. However, all of these policies have failed to decrease binge drinking in both adolescents and adults [33].

In 2010, the European Medicines Agency stated that the main goals of treatment for AUD are to obtain the complete abstinence (“zero-tolerance approach”), the reduction in frequency and severity of relapse and the improvement in health and psychosocial functioning. However, the alternative “harm reduction method” supports a decrease in alcohol consumption and is now considered a possible treatment approach for many patients [34].

The most frequently used psychosocial interventions are comprised of motivational interviewing, cognitive-behavioural therapy, psychodynamic approaches, screening and brief interventions, family therapy, drug counselling, 12-step programs, therapeutic communities and vocational rehabilitation [35]. In particular, motivational interviewing might help patients to evaluate their own personal and clinical condition. Other interesting approaches are brief intervention, which can make possible more healthy behaviors, and cognitive-behavioural therapies that can lead to a decrease of alcohol relapse and craving [36].

All psychosocial interventions seem to be potentially useful for the treatment of AUD. Nevertheless, the available data from the published studies do not permit to establish an universal evidence-based psychological approach [37].

Klimas *et al.* [38] confirmed the above reported suggestion in a specific setting (simultaneous use of alcohol and illicit drug). Through a review article, authors analyzed the feasible psychosocial interventions in the presence of both alcoholism and illicit drug use. Four studies involving 594 participants were considered. The studies reported six different psychosocial interventions, grouped into four comparisons: (1) cognitive-behavioural coping skills training *versus* 12-step facilitation; (2) brief intervention *versus* treatment as usual; (3) group or individual motivational interviewing *versus* hepatitis health promotion; and (4) brief motivational intervention *versus* assessment-only. Authors suggested that the shortage of the data and the low quality of the analyzed studies does not permit drawing any conclusions regarding the best therapeutic psychosocial approach.

The lack of strong data makes it difficult to establish which the best psychological approach is for patients with AUD. Indeed, it would be important to develop randomized controlled trials to examine the effectiveness of this kind of therapy.

## 4. Psychiatric and Pharmacological Management of Alcohol Disorder

During the psychiatric evaluation of patients with AUD, it is mandatory to consider the following complex aspects: type of alcoholic patient (*i.e.*, Cloninger type I and II), variety of craving, gene polymorphism and coexisting co-morbidities. Regarding this last point, AUD is often associated with other mental disorders. In fact, more than one third of people affected by mental disorders show an alcohol-related clinical problem, and, among patients with AUD, 37% suffer from mental illnesses. AUD is connected to an increased risk of mood disorders with respect to the general population. In particular, depression is more than three times higher, bipolar disorder four times higher, anxiety disorders more than six times higher, panic disorders more than four times higher, and post-traumatic stress disorder double the average mood disorders [39].

The main pharmacological choices for patients with AUD are summarized in Table 2. Notably, the Food and Drug Administration have approved only the following drugs: disulfiram (DF), naltrexone (NTX), and acamprosate (ACM), all not fully effective in maintaining abstinence [40]. In addition, in Italy and Austria, sodium oxybate (SMO) is also approved. Nalmefene (NMF) is a drug with opioidergic activity, which was approved in February 2013 by the European Medicines Agency. It was granted market authorization in the European Union for the reduction of alcohol consumption in alcohol-dependent patients with a high drinking risk level (defined as an alcohol intake >60 g/day for men and >40 g/day for women) [41].

**Table 2:** Pharmacological options for patients with AUD.

Drug	Molecular Mechanism	Ideal Indication(s)	Main Side Effect(s)	References
Disulfiram	aldehyde dehydrogenase inhibitor	cocaine users	acetaldehyde syndrome	[42,43]
Naltrexone	opioid receptor antagonist	craving, familiarity, Asn40Asp, OPRM1	headaches, nausea, dyspepsia, anorexia, anxiety, sedation	[44,45]
Acamprosate	*N*-methyl-d-aspartate antagonist	dysphoria, long-term therapy	diarrhea	[46,47]
Sodium Oxybate	GABA-_B_ agonist	no poly-drug addiction, no psychiatric	drug craving and abuse	[48,49,50,51,52]
Nalmefene	opioidergic activator	heavy drinking	insomnia, headache, nausea	[53,54]
Baclofen	GABA-_B_ agonist	craving, ALD	drowsiness, muscle hypotonia	[55,56,57]

DF can permanently inhibit the action of the aldehyde dehydrogenase enzyme leading to accumulation of acetaldehyde consequent to the ethanol intake. The possible consequence is the so called “acetaldehyde syndrome” characterized by heat in the face, purple rash, tachycardia, hypertension, nausea, vomiting, diarrhea, headaches and breath alterations. Indeed, these symptoms appearing in a few minutes represent a strong deterrent for alcohol consumption [42]. Notably, patients with psychotic disorder, severe liver disease, peripheral neuropathy and optic neuritis cannot be treated with this drug. Moreover, due to its inhibitory activity on the dopamine-beta-hydroxylase enzyme, DF increases dopamine concentration with a consequent reduction in cocaine use [43].

NTX is an opioid receptor antagonist, which decreases dopamine release from the limbic system. This drug tends to decrease the amount of alcohol intake in non-abstinent subjects, especially if associated with psychosocial treatments [44]. The typical side effects are headaches, nausea, dyspepsia, anorexia, anxiety and sedation. Interestingly, high levels of craving, a positive family history of alcoholism and the presence of a specific functional polymorphism (Asn40Asp) of the mu-opioid receptor gene (OPRM1), are well known predictors of response to this drug [45].

ACM shows an antagonistic activity on the *N*-methyl-d-aspartate glutamate receptor and a consequent neuro-protective effect [46]. By improving the dysphoria often found in alcoholics, this mechanism indirectly causes a reduction in alcohol craving. According to the meta-analysis by Rösner *et al.* [47], ACM is efficient and safe to support continuous abstinence after detoxification by alcohol. Markedly, diarrhea is the only possible relevant side effect of this drug. SMO is a physiological short-chain fatty acid structurally similar to the inhibitory neurotransmitter γ-amino-butyric acid (GABA) [48]. SMO is located in the mammalian central nervous system, and binds to GABA_B_ receptors exerting an ethanol-mimicking effect [49]. In Europe, SMO is used as an anaesthetic in Germany, and has been used for the treatment of AUD in Italy, since 1992, and in Austria since 1999. Indeed, it has been demonstrated that SMO can be considered a safe and efficient drug for the treatment of AUD, for both the treatment of alcohol withdrawal syndrome and the prevention of alcohol relapse [50]. Concerning the risk of developing addiction to, misuse or abuse of SMO, clinical trials have shown that craving for and abuse of SMO are a very limited phenomenon (~10%), being strictly related to poly-drug addiction and psychiatric comorbidity [51,52].

NMF is a µ and δ-opiod antagonist and κ-opioid partial-agonist, which seems to determine a decrease of heavy drinking. This has been reported by two randomized, double blind, placebo-controlled trials [53,54] where patients with AUD received the drug “as-needed” (defined as self-identified high risk situations).

Several other compounds have been proposed for the treatment of AUD with inconclusive data about efficacy and safety. The chance to use the synergistic effect of different drugs, the characterization of different sorts of craving and AUD, the use of personalized treatments, and the study of genetic polymorphism of receptors targeted by anti-craving molecules, would lead to significant growth of therapeutic success [43].

Baclofen (a GABA-_B_ agonist) represents the only anti-craving medication tested in a randomized controlled clinical trial with subjects affected by advanced alcoholic liver disease (ALD) [55]. This is due to its primary renal metabolism and its low liver metabolism that determine a non-relevant risk of liver damage [56]. Addolorato *et al.* [55] suggested that baclofen was significantly more effective than placebo in inducing and maintaining alcohol abstinence and in increasing CAD in alcohol-dependent patients who were seeking the treatment. Yamini *et al.* [57] reported that baclofen is safe and useful in maintaining alcohol abstinence, favoring the improvement of disease severity too. The guidelines of the European Association for the Study of Liver [5] confirmed the utility of baclofen in patients with advanced ALD.

## 5. Alcoholic Liver Disease

ALD represents the main cause of liver illness in both North America and Europe. The impact of alcohol on liver-related mortality is well known. In the last years, a decline in cirrhosis-related mortality can be found in those countries where alcohol consumption is decreasing such as North America, Australia and Southern Europe. On the contrary, the mortality related to alcohol cirrhosis remains high in the places where an alcohol habit is not declining, such as Hungary [58,59].

Alcohol determines liver damage directly due to oxidative stress, inflammation, and endotoxins [60]. Interestingly, it was reported that the progression of ALD is strictly linked to an increase in intestinal permeability and endotoxemia. In details, ethanol and acetaldehyde have a negative effect on epithelial tight junctions in the intestine, leading to an increased intestinal permeability to endotoxins.

It has to be underlined that there is a dose-response relationship between the amount of alcohol consumed and the risk of ALD development. In particular, the hazard seems to begin at 20–30 g ethanol intake per day, with cirrhosis developing in 10%–20% of patients drinking more than 80 g of ethanol daily [28]. Notably, recent alcohol consumption, rather than earlier in life consumption, is associated with a higher risk of alcoholic cirrhosis [61]. A meta-analysis indicated that consumption of more than 25 g/day increases the relative risk of cirrhosis [62]. The European Association for the Study of the Liver (EASL) stated that there is an increased risk of death from liver cirrhosis in men and women drinking 12–24 g of ethanol per day [5].

It was also reported that consuming alcohol outside of meal times can increase the risk of ALD by 2.7-fold in comparison with people who drink alcohol only during meals [63]. Considering the length of exposure, in heavy drinkers, the risk of cirrhosis is 50% at 61 years of age, with a shorter time of progression to cirrhosis in women [64].

The spectrum of ALD includes simple steatosis (rapidly reversible), alcoholic steatohepatitis (infiltration of polymorphonuclear cells, hepatocyte ballooning and Mallory-Denk’s bodies), progressive fibrosis, cirrhosis and the development of hepatocellular carcinoma (HCC). Although the risk of HCC in alcoholic cirrhosis is lower in comparison with viral cirrhosis, it has to be taken into account that it also remains, even if inferior, in the case of abstinence [65].

ALD progression varies according to both genetic and environmental factors. The main relation of people with a genetic predisposition to develop ALD is supported by the observation of concordance rates for AUD and alcohol-induced liver fibrosis in monozygotic twins of 26.3 and 14.6 [66].

Although simple steatosis is clinically silent and reversible, it seems to affect the long-term survival. In fact, the ten-year mortality of patients with alcoholic steatosis was significantly higher with respect to non-alcoholic fatty liver disease (74% *versus* 25%) and more subjects developed cirrhosis (21% *versus* 1%) [67]. Patients with underlying severe fibrosis or cirrhosis and heavy alcohol intake can present a form of acute-on-chronic liver failure called alcoholic hepatitis (AH), characterized by a rapid liver function worsening [68]. Notably, patients with severe forms of AH show high short-term mortality (30%–50%). Patients with AH disclosed the fastest progression of fibrosis and, consequently, an increased risk of liver-related death [69]. Among other factors that can influence the outcome of patients with ALD determining a fastest disease progression, there is obesity. In fact, heavy drinkers who are overweight for at least 10 years have a twofold risk of developing cirrhosis [70]. This fact has two explanations: (a) obesity predisposes to alcohol-induced fatty liver; (b) obesity favors the development of AH since insulin resistance seems to worsen the effects of alcohol in the liver tissue. As a consequence, it was reported that hyperglycaemia is an independent predictor of progression of fibrosis in heavy drinkers [71].

Since many patients with AUD are polysubstance users, parenteral virus infections such as hepatitis B and C, can be found. Interestingly, patients with AUD exhibit a high prevalence of viral infection in the absence of polysubstance use and abuse, being at high risk of trauma, hospitalization, blood transfusions, and risky sexual behaviour. However, alcohol habit negatively affects viral liver disease by suppressing Major Histocompatibility Complex-I and II and by direct and indirect negative effects on viral replication, increasing both oxidative stress and cytotoxicity, and weakening immune response. All these actions together negatively affect prognosis of these patients, leading to accelerated progression of liver damage, a superior incidence of HCC, and, definitively, worst prognosis in terms of morbidity and mortality. Moreover, the consolidated drugs for hepatitis B and the new drugs for hepatitis C are emerging with very good therapeutic success. In this context, alcohol use might lead to lack of compliance with the consequent risk of losing the chance of antiviral treatment [28].

## 6. The Multidisciplinary Approach in the Transplantation Field

Alcoholic cirrhosis is a leading reason of end-stage liver disease and/or HCC and the second most common indication for LT in the Western world [72]. Many authors suggested that end-stage ALD is a good indication for LT seen the great graft and patient survival and the little percentage of clinically relevant alcohol relapse [73,74]. The organ donation shortage makes transplant accessible only to a minor part of needing patients and many people consider patients with AUD unworthy for the access to the waiting list. Maybe also for this reason, widely accepted disease-specific selection criteria exist for the majority of indications, but the selection of alcoholic candidates remains controversial [75]. Most transplants routinely require six months of documented zero alcohol consumption before the entrance in the waiting list (“six-month rule”) [76].

Although there is evidence that a shorter pre-LT abstinence period corresponds to a shorter relapse after LT, its ideal length is still debatable [77].

The “Group of Italian Regions” (Department of Health), on the basis of a comprehensive review of the published literature and, with particular regard to the daily clinical practice of those who have participated in the workshop “Alcohol and Liver Transplant”, affirms that the “six-month rule” is not an evidence-based practice [78].

In particular, there are many other factors that can influence the risk of relapse and the compliance of these patients. In this regard, the individual multidisciplinary evaluation becomes central mostly in the pre-transplant phase. In fact, patients who have good social support, who do not suffer from psychotic or personality disorders and who have decompensated liver disease, should be added to the liver transplant list, without taking the length of their period of abstinence into consideration showing a very low probability to relapse [79]. Indeed, the framing of the candidates by social and psychological/psychiatric point of view, their training about transplants in terms of information and psychological intervention, the building or the strengthening of social and familiar support, are essential. In the clinical practice, before the transplant, hepatologists have to exclude the presence of contraindications for the transplant. Psychologists should preliminarily introduce the patients in the new world represented by the waiting list and evaluate his personality and support. Psychiatrists have to exclude psychotic disorders and assess the use of targeted drugs.

After LT, a new psychological and clinical phase starts. In that period, patients with other minor medical conditions and who have strong social support, should be offered cognitive behavioral therapy, while patients with significant other medical conditions and/or limited social support should be allowed into specific programs including multi-dimensional family therapy, functional family therapy and brief strategic family therapy [80]. Markedly, attendance and active participation to self-help group (for example, Alcoholics Anonymous) might be helpful for all patients with AUD in both pre and post-LT period [81].

Notably, according to the recently published “Italian position paper” [82], we agree with the following points: (1) in cases of cirrhosis with MELD <19, six months of abstinence are opportune and useful to understand if a recover of liver function can be possible; (2) in cases of progressive ALD with MELD >19, three months of abstinence seem to be more appropriate in patients with an adequate care-giver support, into a psychosocial program and without an associated severe psychiatric disorder; (3) in cases of severe alcoholic hepatitis not responding to medical therapy (Maddrey score > 32 or MELD >21), LT should be accurately considered as a life-saving therapeutic option in well selected patients (see point 2) also without the achievement of three or six months of abstinence; (4) the multidisciplinary transplant team must include clinical psychologists, psychiatrists and hepato-alcohologists; and (5) patients should be followed in all phases of the complex transplant road also with the help of self-help groups.

## 7. Conclusions

Alcohol represents at the same time a relevant part of people’s ordinary lives and a significant cause of both morbidity and mortality worldwide. The AUD has relevant negative economic impact on the society and favors violence, accidents and loss in quality of life of both patients and family members.

Although an evidence-based psychosocial approach is not available, it is reasonable to assert that it should be a central part of the management of patients with AUD. In fact, the psychiatric and pharmaceutical approaches are not definitive showing some good effects but also relevant limits. Many drugs are accessible for the treatment of AUD, but Baclofen represents the only anti-craving medication useful also in advanced ALD. ALD represents the most frequent cause of advanced liver disease in Europe, being a leading cause of death among adults with excessive alcohol consumption.

In the management of ALD patients, the social support and the multidisciplinary action of clinical psychologists, psychiatrists and hepato-alcohologists, are mandatory to achieve the therapeutic success, especially in the field of LT. In fact, in the pre-LT phase, patients often have to bear a long waiting time and a complex psychological assessment. Moreover, in the post-period, patients can consider their status as a sort of never-ending disease, and, also for this reason, the maintenance of abstinence can be difficult. In this context, the rigidity of the transplant system and the adoption of the six-month rule are at least questionable.

In conclusion, the multidisciplinary approach is essential. In the pre-alcohol period of life, it is important for the sensitization of public health and the prevention, during the first phase of AUD for the correct framing of patients and in the last part of the illness course, for the management of all the LT phases.

The Health Agencies and the Governments should develop population-based policies for decreasing the prevalence of harmful alcohol intake and for endorsing the research in this field. Major information, a growing awareness and an improvement in the health care knowledge of people, might be the keys to reduce this major health and social problem.

## References

[1] Maher J.J., Feldman M., Friedman L.S., Sleisenger M.H. (2002). Alcoholic liver disease. Gastrointestinal and Liver Disease.

[2] Cochrane J., Chen H., Conigrave K.M., Hao W. (2003). Alcohol use in China. Alcohol Alcohol..

[3] Anderson P., Baumberg B. (2006). Alcohol in Europe: A Public Health Perspective.

[4] Wittchen H.U., Jacobi F. (2005). Size and burden of mental disorders in Europe—A critical review and appraisal of 27 studies. Eur. Neuropsychopharm..

[5] European Association for the Study of Liver (2012). EASL clinical practical guidelines: Management of alcoholic liver disease. J. Hepatol..

[6] What Is a Standard Drink?. www.niaaa.nih.gov/alcohol-health/overview-alcohol-consumption/standard-drink.

[7] Bagnardi V., Rota M., Botteri E., Tramacere I., Islami F., Fedirko V., Scotti L., Jenab M., Turati F., Pasquali E. (2013). Light alcohol drinking and cancer: A meta-analysis. Ann. Oncol..

[8] Laramée P., Kusel J., Leonard S., Aubin H.J., François C., Daeppen J.B. (2013). The economic burden of alcohol dependence in Europe. Alcohol Alcohol..

[9] Mohapatra S., Patra J., Popova S., Duhing A., Rehm J. (2010). Social cost of heavy drinking and alcohol dependence in high-income countries. Int. J. Public Health.

[10] American Psychiatric Association (2013). Diagnostic and Statistical Manual of Mental Disorders.

[11] Leggio L., Kenna G.A., Fenton M., Bonenfant E., Swift R.M. (2009). Typologies of alcohol dependence. From Jellinek to genetics and beyond. Neuropsychol. Rev..

[12] Johnson B.A. (2010). Medication treatment of different types of alcoholism. Am. J. Psychiatry.

[13] Pombo S., Lesch O.M. (2009). The alcoholic phenotypes among different multidimensional typologies: Similarities and their classification procedures. Alcohol Alcohol..

[14] Cloninger C.R., Bohman M., Sigvardsson S. (1981). Inheritance of alcohol abuse. Cross-fostering analysis of adopted men. Arch. Gen. Psych..

[15] Babor T.F., Hofmann M., del Boca F.K., Hesselbrock V., Meyer R.E., Dolinsky Z.S., Rounsaville B. (1992). Types of alcoholics, I. Evidence for an empirically derived typology based on indicator of vulnerability and severity. Arch. Gen. Psychiatry.

[16] Harnic D., Digiacomantonio V., Innamorati M., Mazza M., di Marzo S., Sacripanti F., Saioni R., Cardella A., di Felice C., Girardi P. (2010). Temperament and attachment in alcohol addicted patients of type 1 and 2. Riv. Psichiatr..

[17] Cibin M., Spolaor G., Hinnental I., Sgualdini E., Chiamulera C. (2015). Residnetial treatment of alcohol use disorders: Thinking about the future. Alcologia.

[18] Sinha R., O’Malley S.S. (1999). Craving for alcohol: Findings from the clinic and the laboratory. Alcohol Alcohol..

[19] Addolorato G., Abenavoli L., Leggio L., Gasbarrini G. (2005). How many cravings? Pharmacological aspects of craving treatment in alcohol addiction: A review. Neuropsychobiology.

[20] Addolorato G., Leggio L., Abenavoli L., Gasbarrini G. (2005). Alcoholism Treatment Study Group. Neurobiochemical and clinical aspects of craving in alcohol addiction: A review. Addict. Behav..

[21] Martinotti G., di Nicola M., Tedeschi D., Callea A., di Giannantonio M., Janiri L., Craving Study Group (2013). Craving Typology Questionnaire (CTQ): A scale for alcohol craving in normal controls and alcoholics. Compr Psychiatry.

[22] Zakhari S., Li T. (2007). Determinants of alcohol use and abuse: Impact of quantity and frequency patterns on liver disease. Hepatology.

[23] Pincock S. (2003). Binge drinking on rise in UK and elsewhere. Lancet.

[24] Naimi T.S., Brewer R., Mokdad A., Denny C., Serdula M.K., Marks J.S. (2003). Binge drinking among US adult. JAMA.

[25] Mathurin P., Delterne P. (2009). Effect of binge drinking on the liver: An alarming public health issue?. Gut.

[26] Litten R.Z., Egli M., Heilig M., Cui C., Fertig J.B., Ryan M.L., Falk D.E., Moss H., Huebner R., Noronha A. (2012). Medications development to treat alcohol dependence: A vision for the next decade. Addict. Biol..

[27] Neuberger J., Adams D., MacMaster P., Maidment A., Speed M. (1998). Assessing priorities for allocation of donor liver grafts: Survey of and clinicians. BMJ.

[28] Gitto S., Vitale G., Villa E., Andreone P. (2014). Update on Alcohol and Viral Hepatitis. J. Clin. Transl. Hepatol..

[29] Samokhvalov A.V., Popova S., Room R., Ramonas M., Rehm J. (2010). Disability associated with alcohol abuse and dependence. Alcohol. Clin. Exp. Res..

[30] Mathurin P., Bataller R. (2015). Trends in the management and burden of alcoholic liver disease. J. Hepatol..

[31] Singh V., Epstein-Ngo Q., Cunningham R.M., Stoddard S.A., Chermack S.T., Walton M.A. (2015). Physical dating violence among adolescents and young adults with alcohol misuse. Drug Alcohol Depend..

[32] Leon D.A., Mc Cambridge J. (2006). Liver cirrhosis mortality rates in Britain from 1950 to 2002: An analysis of routine data. Lancet.

[33] Wagenaar A.C., Tobler A.L., Komro K.A. (2010). Effects of alcohol tax and price policies on morbidity and mortality: A systematic review. Am. J. Public Health.

[34] Guideline on the Development of Medicinal Products for the Treatment of Alcohol Dependence. http://www.ema.europa.eu/docs/en_GB/document_library/Scientific_guideline/2010/03/WC50004898.pdf.

[35] Amato L., Minozzi S., Davoli M., Vecchi S. (2011). Psychosocial and pharmacological treatments *versus* pharmacological treatments for opioid detoxification. Cochrane Database Syst. Rev..

[36] Kaner E.F., Beyer F., Dickinson H.O., Pienaar E., Campbell F., Schlesinger C., Heather N., Saunders J., Burnand B. (2007). Effectiveness of brief alcohol interventions in primary care populations. Cochrane Database Syst. Rev..

[37] Whitlock E.P., Polen M.R., Green C.A., Orleans T., Klein J. (2004). Behavioural counselling interventions in primary care to reduce risky/harmful alcohol use by adults: A summary of the evidence for the U.S. Preventive services task force. Ann. Intern. Med..

[38] Klimas J., Tobin H., Field C.A., O’Gorman C.S., Glynn L.G., Keenan E., Saunders J., Bury G., Dunne C., Cullen W. (2014). Psychosocial interventions to reduce alcohol consumption in concurrent problem alcohol and illicit drug users. Cochrane Database Syst. Rev..

[39] Klimkiewicz A., Klimkiewicz J., Jakubczyk A., Kieres-Salomoński I., Wojnar M. (2015). Comorbidity of alcohol dependence with other psychiatric disorders. Part I. Epidemiology of dual diagnosis. Psychiatr. Pol..

[40] Krampe H., Ehrenreich H. (2010). Supervised disulfiram as adjunct to psychotherapy in alcoholism treatment. Curr. Pharm. Des..

[41] European Public Assessment Reports. http://www.ema.europa.eu/ema/index.jsp?curl=pages/medicines/landing/epar_search.jsp&mid=WC0b01ac058001d125.

[42] Chick J. (1999). Safety issues concerning the use of disulfiram in treating alcohol dependence. Drug Saf..

[43] Caputo F., Vignoli T., Grignaschi A., Cibin M., Addolorato G., Bernardi M. (2014). Pharmacological management of alcohol dependence: From mono-therapy to pharmacogenetics and beyond. Eur. Neuropsychopharmacol..

[44] Rösner S., Hackl-herrwerth A., Leucht S., Vecchi S., Srisurapanont M., Soyka M. (2010). Opioid antagonists for alcohol dependence. Cochrane Database Syst. Rev..

[45] Anton R.F., Oroszi G., O’Malley S., Couper D., Swift R., Pettinati H., Goldman D. (2008). An evaluation of muopioid receptor (OPRM1) as a predictor of naltrexone response in the treatment of alcohol dependence: Results from the combined pharmacotherapies and behavioural interventions for alcohol dependence (COMBINE) study. Arch. Gen. Psychiatry.

[46] De Witte P., Littleton J., Parot P., Koob G. (2005). Neuroprotective and abstinence-promoting effects of acamprosate: Elucidating the mechanism of action. CNS Drugs.

[47] Rösner S., Hackl-herrwerth A., Leucht S., Lehert P., Vecchi S., Soyka M. (2010). Acamprosate for alcohol dependence. Cochrane Database Syst. Rev..

[48] Snead O.C., Gibson K.M. (2005). Gamma-hydroxybutyric acid. N. Engl. J. Med..

[49] Caputo F., Bernardi M. (2010). Medications acting on the GABA system in the treatment of alcoholic patients. Curr. Pharm. Des..

[50] Leone M.A., Vigna-Taglianti F., Avanzi G., Brambilla R., Faggiano F. (2010). Gamma-hydroxybutyrate (GHB) for treatment of alcohol withdrawal and prevention of relapses. Cochrane Database Syst. Rev..

[51] Keating G.M. (2014). Sodium oxybate: A review of its use in alcohol withdrawal syndrome and in the maintenance of abstinence in alcohol dependence. Clin. Drug Investig..

[52] Skala K., Caputo F., Mirijello A., Vassallo G., Antonelli M., Ferrulli A., Walter H., Lesch O., Addolorato G. (2014). Sodium oxybate in the treatment of alcohol dependence: From the alcohol withdrawal syndrome to the alcohol relapse prevention. Expert Opin. Pharmacother..

[53] Mann K., Bladström A., Torup L., Gual A., Van den Brink W. (2013). Extending the treatment options in alcohol dependence: A randomized controlled study of as-needed nalmefene. Biol. Psychiatry.

[54] Gual A., Heb Y., Torup L., van den Brink V., Mann K., ESENSE 2 study group (2013). A randomised, double-blind, placebo-controlled, efficacy study of nalmefene, as-needed use, in patients with alcohol dependence. Eur. Neuropsychopharmacol..

[55] Addolorato G., Leggio L., Ferrulli A., Cardone S., Vonghia L., Mirijello A., Abenavoli L., D’Angelo C., Caputo F., Zambon A. (2007). Effectiveness and safety of baclofen for maintenance of alcohol abstinence in alcohol-dependent patients with liver cirrhosis: Randomised, double-blind controlled study. Lancet.

[56] Addolorato G., Leggio L., Agabio R., Colombo G., Gasbarrini G. (2006). Baclofen: A new drug for the treatment of alcohol dependence. Int. J. Clin. Pract..

[57] Yamini D., Lee S.H., Avanesyan A., Walter M., Runyon B. (2014). Utilization of baclofen in maintenance of alcohol abstinence in aptients with alcohol dependence and alcohol hepatitis with or without cirrhosis. Alcohol Alcohol..

[58] Corrao G., Ferrari P., Zambon A., Torchio P., Arico S., Decarli A. (1997). Trends of liver cirrhosis mortality in Europe, 1970–1989: Age-period-cohort analysis and changing alcohol consumption. Int. J. Epidemiol..

[59] Ramstedt M. (2001). Per capita alcohol consumption and liver cirrhosis mortality in 14 European countries. Addiction.

[60] Testino G. (2008). Alcoholic diseases in hepato-gastroenterology: A point of view. Hepatogastroenterology.

[61] Askgaard G., Gronbaek M., Kjaer M.S. (2015). Alcohol drinking pattern and risk of alcoholic liver cirrhosis: A prospective cohort study. J. Hepatol..

[62] Corrao G., Bagnardi V., Zambon A., Torchio P. (1998). Meta-analysis of alcohol intake in relation to risk of liver cirrhosis. Alcohol Alcohol..

[63] Mathurin P., Lucey M.R. (2012). Management of alcoholic hepatitis. J. Hepatol..

[64] Testino G. (2013). Alcoholic hepatitis. J. Med. Life.

[65] Poynard T., Mathurin P., Lai C.L., Guyader D., Poupon R., Tainturier M.H., Myers R.P., Muntenau M., Ratziu V., Manns M. (2003). A comparison of fibrosis progression in chronic liver diseases. J. Hepatol..

[66] Hrubec Z., Omenn G.S. (1981). Evidence of genetic predisposition to alcoholic cirrhosis and psychosis: Twin concordances for alcoholism and its biological end points by zygosity among male veterans. Alcohol. Clin. Exp. Res..

[67] Liu Y.L., Patman G.L., Leathart J.B., Piguet A.C., Burt A.D., Dufour J.F., Day C.P., Daly A.K., Reeves H.L., Anstee Q.M. (2014). Carriage of the PNPLA3 rs738409 C >G polymorphism confers an increased risk of non-alcoholic fatty liver disease associated hepatocellular carcinoma. J. Hepatol..

[68] Dam-Larsen S., Franzmann M., Andersen I.B., Christoffersen P., Jensen L.B., Sørensen T.I., Becker U., Bendtsen F. (2004). Long term prognosis of fatty liver: Risk of chronic liver disease and death. Gut.

[69] Mathurin P., Beuzin F., Louvet A., Carrie-Ganne N., Balian A., Trinchet J.C., Dalsoglio D., Prevot S., Naveau S. (2007). Fibrosis progression occurs in a subgroup of heavy drinkers with typical histological features. Aliment. Pharmacol. Ther..

[70] Naveau S., Giraud V., Borotto E., Aubert A., Capron F., Chaput J.C. (1997). Excess weight risk factor for alcoholic liver disease. Hepatology.

[71] Raynard B., Balian A., Fallik D., Capron F., Bedossa P., Chaput J.C., Naveau S. (2002). Risk factors of fibrosis in alcohol-induced liver disease. Hepatology.

[72] Lucey M.R., Schaubel D.E., Guidinger M.K., Tome S., Merion R.M. (2009). Effect of alcoholic liver disease and hepatitis C infection on waiting list and posttransplant mortality and trans-plant survival benefit. Hepatology.

[73] Biselli M., Gramenzi A., del Gaudio M., Ravaioli M., Vitale G., Gitto S., Grazi G.L., Pinna A.D., Andreone P., Bernardi M. (2010). Long term follow-up and outcome of liver transplantation for alcoholic liver disease: A single center case-control study. J. Clin. Gastroenterol..

[74] Pfitzmann R., Schwenzer J., Rayes N., Seehofer D., Neuhaus R., Nüssler N.C. (2007). Long-term survival and predictors of relapse after orthotopic liver transplantation for alcoholic liver disease. Liver Transpl..

[75] Gaglio P.J., Gaglio P.J. (2012). Complications in patients with alcohol- associated liver disease who undergo liver transplantation. Clin. Liver Dis..

[76] Leong J., Im G.Y. (2012). Evaluation and selection of the patient with alcoholic liver disease for liver transplant. Clin. Liver Dis..

[77] Varma V., Webb K., Mirza D.F. (2010). Liver transplantation for alco-holic liver disease. World J. Gastroenterol..

[78] Regione Autonoma Friuli Venezia Giulia. http://www.mediofriuli.it.

[79] Testino G., Sumberaz A., Borro P. (2013). Comment to “liver transplantation for patients with alcoholic liver disease: An open question”. Dig. Liver Dis..

[80] Pilling S., Yesufu-Udechuku A., Taylor C., Drummond C. (2011). Diagnosis, assessment, and management of harmful drinking and alcohol dependence: Summary of NICE guidance. BMJ.

[81] Curzio O., Tilli A., Mezzasalma L., Scalese M., Fortunato L., Potente R., Guidoni G., Molinaro S. (2012). Characteristics of alcoholics attending “clubs of alcoholics in treatment” in Italy: A national survey. Alcohol Alcohol..

[82] Testino G., Burra P., Bonino F., Piani F., Sumberaz A., Peressutti R., Giannelli Castiglione A., Patussi V., Fanucchi T., Ancarani O. (2014). Acute alcoholic hepatitis, end stage alcoholic liver disease and liver transplantation: An Italian position statement. World J. Gastroenterol..

